# Blood pressure and cardiovascular risk in a contemporary cohort of young adults

**DOI:** 10.1093/ajh/hpag025

**Published:** 2026-03-25

**Authors:** Eirik Aaseth, Sigrun Halvorsen, Sverre Erik Kjeldsen, Jørgen Gravning

**Affiliations:** Department of Cardiology, Oslo University Hospital Ullevål, Oslo, Norway; Institute of Clinical Medicine, University of Oslo, Oslo, Norway; Department of Cardiology, Oslo University Hospital Ullevål, Oslo, Norway; Institute of Clinical Medicine, University of Oslo, Oslo, Norway; Department of Cardiology, Oslo University Hospital Ullevål, Oslo, Norway; Institute of Clinical Medicine, University of Oslo, Oslo, Norway; Department of Cardiology, Oslo University Hospital Ullevål, Oslo, Norway; Institute of Clinical Medicine, University of Oslo, Oslo, Norway

**Keywords:** blood pressure, cardiovascular disease, contemporary study, hypertension, young adults

## Abstract

**Background:**

Previous studies suggest that blood pressure (BP) is a stronger risk factor for cardiovascular disease (CVD) in women compared to men. However, few have rigorously investigated this association in young adults with emphasis on sex differences.

**Methods:**

In 2000, the “Oslo Health Study” (HUBRO), recruited 5933 individuals born in 1969 or 1970. By linkage with national health registries in Norway, we followed this cohort through 2024 with respect to CVD events. Multivariate Cox analyses were performed to assess the risk of CVD during follow-up. Sex-specific analyses were performed by interaction testing.

**Results:**

The median age at baseline was 31 years (interquartile range (IQR): 30-31), and 56% were women. The median systolic and diastolic BP was 122 (IQR 114-130) and 69 (IQR 63-75) mm Hg, respectively. After 24 years of follow-up, 158 (2.7%) CVD events had occurred. The adjusted hazard ratio (HR) (95% confidence interval) for CVD events per 10 mm Hg higher systolic and 5 mm Hg higher diastolic BP was 1.22 (1.04-1.42) and 1.20 (1.09-1.31), respectively. The HR per 10 mm Hg higher systolic BP was 1.30 (1.01-1.68) in women vs 1.15 (0.96-1.38) in men. No statistically significant sex-interaction between systolic BP and risk of CVD event was found (*P* = .38). The HR per 5 mm Hg higher diastolic BP was 1.20 (1.03-1.41) in women and 1.20 (1.07-1.34) in men.

**Conclusions:**

In young adults from the general population, systolic and diastolic BP were related to the risk of CVD events during long-term follow-up, with similar risk in women and men.

## Graphical abstract

**Graphical Abstract:**
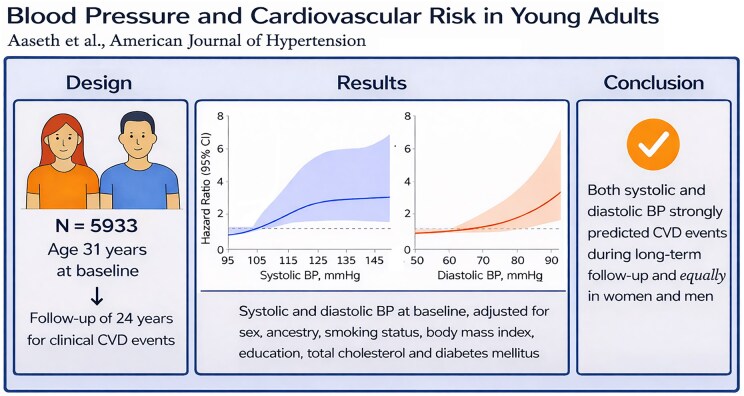

## Introduction

Most epidemiological and therapeutic studies on hypertension have primarily focused on middle-aged or older adults at high risk of cardiovascular disease (CVD).^1^ In contrast, young adults (<40 years of age) typically have a low 10-year risk of CVD events despite a potentially high lifetime risk. Previous studies of blood pressure (BP) as a long-term CVD risk factor in young adults are largely historical or have been conducted in selected populations.^2^ Studies of Swedish male military conscripts have shown that BP at age 18 years is associated with long-term mortality and major CVD events, with risk gradually increasing from BP levels around 120/80 mm Hg.^3^^,^^4^ These findings were limited to a selected male population and are not representative of the general population. Similar studies in young women are scarce; however, a Norwegian study reported that BP was a stronger CVD risk factor in middle-aged women than in men (with risk emerging at a lower BP nadir).^5^ Other studies have also reported similar findings, thereby proposing sex differences in BP pathophysiology.^6-9^ However, meta-analyses conducted mainly in middle-aged and older adults have not reported a higher risk for the relationship of BP with CVD events in women.^10^^,^^11^ Thus, further research is needed to understand potential sex differences of BP on the risk of CVD events, especially in young adults.

Of concern, the incidence rates of myocardial infarction, stroke, and chronic kidney disease among young adults in the US are increasing.^12^ Although the prevalence of hypertension in young adults in the US has remained stable, control rates have worsened over the past decades compared with older age groups.^13^ In contrast, health surveys from Western Europe, including participants from the general population, have shown decreasing average BP during the same period.^14^^,^^15^ Simultaneously, the incidence rates of myocardial infarction have decreased substantially. However, this beneficial trend has been less pronounced among young adults.^16^ Together, these observations underscore the need for updated knowledge on early risk factor exposure and the long-term risk of CVD.

Consequently, our present study aimed to investigate the relationship between systolic and diastolic BP on the long-term risk of clinical CVD events in a contemporary cohort of young adults recruited from the general population. We also tested the hypothesis of sex-specific associations between BP and future CVD events in our population of young adults in the lifespan toward middle age.

## Methods

### Study population

This prospective cohort study was initiated in the year 2000, as the “Oslo Health Study” (HUBRO). All individuals born in 1969 and 1970 who were living in the city of Oslo, Norway, were invited to participate. The main outline has been described previously.^17^ Of 22,326 persons invited from the general population of 30-year-old women and men, 6300 (28%) participated in the initial examination, forming the basis for this cohort study.^17^ Participants with the highest risk profile based on pre-specified criteria were referred to Oslo University Hospital for further follow-up (*N* = 48). In the present study, participants without recorded BP measurements (*n* = 17), participants with self-reported previous CVD events at baseline (*n* = 16), and participants without a Norwegian social security number (*n* = 334) were excluded. The study is approved by the Regional Committees for Medical and Health Research Ethics in Norway (REK south-east, #265625). All participants provided written informed consent. The first author had full access to all the data in the study and takes responsibility for its integrity and the data analysis.

### Clinical information

Sex, education, past medical history, medication use, and smoking habits, as well as the birthplace of participants and their parents, were self-reported through the study questionnaire. Non-Western ancestry was defined as birth outside Western Europe or North America for the participant or their mother. Non-fasting blood samples were analyzed for serum glucose, total cholesterol, HDL cholesterol, and plasma triglycerides. The presence of diabetes mellitus was defined through self-reporting or by a non-fasting serum glucose >11 mmol/L.

### BP measurements

After sitting in a quiet room for 2 minutes’ rest, BP and heart rate were measured with a validated semi-automatic oscillometric device (Dinamap, Criticon, Tampa, FL, USA) with a suitable cuff size. BP was measured in the seated position 3 times by trained medical personnel with a 1-minute interval, and the average of 2 and 3 measurements was used for follow-up.

### Follow-up

All Norwegians are identified with a unique national identity number. Follow-up data were obtained through linkage of their personal identification number to mandatory nationwide health registries of Norway. Non-fatal myocardial infarction was defined by International Classification of Disease (ICD)-10 codes of I21-I22, stroke by I63-I64 or I67, cerebral hemorrhage by I60-I61, and hospitalization for heart failure by I50 (Table S1). Coronary revascularization was defined by codes using the Norwegian classification system including both surgical and percutaneous interventions linked to ICD-10 codes I20 (angina) or I25 (atherosclerotic heart disease). Only hospitalized patients with one of the abovementioned codes as the first diagnosis were included, except for coronary revascularization, for which outpatient procedures were allowed. As cardiac death, we included sudden death of unknown cause (ICD-10: R99) and cardiac arrest (I46) in addition to the other codes listed in Table S1 if these were one of the first two codes on the death certificate.

The completeness of the national health registries in Norway is high,^18^ and the validity of the diagnosis of myocardial infarction and ischemic stroke is reported to be high.^18-20^

### Outcomes

The cardiovascular outcome was defined as a composite of cardiovascular death, non-fatal myocardial infarction, non-fatal ischemic stroke, non-fatal cerebral hemorrhage, hospitalization for heart failure, and coronary revascularization for other causes than myocardial infarction.

### BP classification

Participants were stratified into 3 BP levels: Level 1 included participants with systolic BP less than 120 mm Hg and diastolic BP less than 70 mm Hg, in accordance with “non-elevated BP” by the European Society of Cardiology.^21^ Level 2 included participants with systolic BP 120-129 mm Hg or diastolic BP 70-79 mm Hg. Level 3 included participants with systolic BP of 130 mm Hg or more or diastolic BP of 80 mm Hg or more in accordance with “grade 1 hypertension” by the American College of Cardiology/American Heart Association or “the elevated BP group suitable for pharmacological treatment when CVD risk is high,” by the European Society of Cardiology.^21^^,^^22^

### Statistical analyses

Demographic data are reported as medians (interquartile range, IQR) and categorical data as numbers (%). Follow-up time was calculated as the interval between the clinical visit at baseline and the first CVD event or throughout 2024 for participants without a CVD event. Individuals who died of non-CVD causes (*n* = 95) were censored at the time of death.

First, associations between BP values on continuous scales and CVD events were tested in Cox regression analyses, adjusted for sex, non-western ancestry, education of less than 12 years, smoking status, diabetes mellitus, body mass index, and total cholesterol. Systolic BP and diastolic BP were tested separately with increments of 10 and 5 mm Hg, respectively. Schoenfeld residuals and log-log plots of event curves were used to test the proportional hazard assumptions, and there were no violations. Restricted cubic splines were plotted to visualize the multivariate adjusted hazard ratios (HRs) for systolic and diastolic BP in relation to CVD events. Akaike information criterion was used to define the number of knots in the plots, and 3 knots were placed at default locations. The reference was set at the 5th percentile, which was 105 and 56 mm Hg for systolic and diastolic BP, respectively.

Second, participants were grouped into BP categories, and cumulative incidence functions were estimated using the Fine-Gray method with non-CVD death as a competing risk. Differences between groups were assessed using Gray’s test. Associations between BP groups and CVD events were estimated with Cox regression analyses with level 1 as the reference. The models were adjusted for the same covariables as above.

Third, associations of BP in relation to CVD events were tested in women and men separately. Restricted cubic splines of the HR and density plots of BP distributions were collectively plotted to visualize potential sex differences with the same procedure as above. Sex differences in BP values were tested using linear regression analyses. To test for interactions between systolic and diastolic BP and sex, multivariate Cox regression models with and without interaction terms were investigated using the likelihood-ratio test.

Fourth, we included both systolic and diastolic BP in the multivariate Cox model to explore their interdependence as risk predictors of CVD events. We performed analyses of the receiver operating curves (ROCs) for each multivariate model, including systolic or diastolic BP, respectively, and tested which component of BP yielded the best discrimination with the DeLong test.

Through sensitivity analyses, we adjusted the Cox models for variables known to influence BP, although they were not statistically significant in univariate or multivariate analyses: Self-reported alcohol consumption (measured as grams per week), self-reported habits of physical activity (measured in 4 categories), the use of antihypertensive medication at baseline, and heart rate.

We also excluded individuals with missing values and reported the results according to blood pressure categories based on the American College of Cardiology/American Heart Association classification, further stratifying Stage 1 hypertension into isolated systolic, isolated diastolic, and systolic-diastolic hypertension.

Missing data for covariates (*n* = 85 for origin, *n* = 104 for education, *n* = 52 for smoking status, *n* = 9 for body mass index, *n* = 9 for total cholesterol) were handled using multiple imputation by chained equations over 20 iterations. All variables were included in the regression models, except for the drawing of splines and ROC curves, for which we used the first imputed dataset. We used R, version 4.3.0 for Windows 64-bit for statistical computing, and the two-sided significance level was set at *P* < .05.

## Results

Our study comprised 5933 participants recruited from the general population in Oslo, Norway, of whom 3308 (56%) were women (Figure S1). The median (IQR) age was 31 (30-31) years. The median systolic and diastolic BP were 122 (114-130) and 69 (63-75) mm Hg, respectively. A total of 1980 (33%) individuals had BP <120/70 mm Hg, and 70 individuals (1%) reported the use of antihypertensive medications at inclusion. Baseline characteristics according to BP groups and for the total population are presented in Table 1.

**Table 1:** Baseline characteristics of the population overall and stratified by categories of blood pressure.

	Overall	Level 1 (SBP < 120 and DBP < 70)	Level 2 (SBP 120-129 or DBP 70-79)	Level 3 (SBP ≥ 130 or DBP ≥ 80)
Variables	*n* = 5933	*n* = 1980	*n* = 2212	*n* = 1741
**Women, *n* (%)**	3308	1595 (81)	1199 (54)	514 (30)
**Age, years**	31 (30-31)	31 (30-31)	31 (30-31)	31 (30-31)
**Non-Western ancestry, *n* (%)**	812 (14)	346 (18)	308 (14)	158 (9)
**Current smokers, *n* (%)**	1381 (23)	453 (23)	521 (24)	407 (24)
**Diabetes, *n* (%)**	44 (1)	16 (1)	16 (1)	12 (1)
**Education, years**	16 (13-18)	16 (13-18)	16 (13-18)	16 (13-18)
**Body mass index, kg/m^2^**	24 (22-27)	23 (21-25)	24 (22-26)	26 (23-28)
**Systolic BP, mm Hg**	122 (114-130)	112 (107-115)	123 (120-126)	135 (131-141)
**Diastolic BP, mm Hg**	69 (63-75)	62 (59-66)	71 (67-74)	78 (72-82)
**Use of antihypertensives, *n* (%)**	70 (1)	21 (1)	23 (1)	26 (2)
**Total cholesterol, mmol/L**	4.9 (4.3-5.6)	4.8 (4.2-5.4)	4.9 (4.4-5.6)	5.1 (4.6-5.9)
**LDL cholesterol, mmol/L**	2.9 (2.4-3.5)	2.8 (2.3-3.3)	2.9 (2.5-3.5)	3.1 (2.6-3.7)
**HDL cholesterol, mmol/L**	1.4 (1.2-1.7)	1.5 (1.3-1.8)	1.4 (1.2-1.7)	1.3 (1.1-1.5)
**Plasma triglycerides, mmol/L**	1.1 (0.81.7)	0.9 (0.7-1.3)	1.1 (0.8-1.7)	1.4 (1.0-2.2)
**No traditional risk factors^a^, *n* (%)**	2169 (37)	927 (47)	799 (36)	443 (26)

Values are median (interquartile range) for continuous variables and number (%) for categorical variables.

Abbreviations: BP, blood pressure; HDL, high-density lipoprotein; LDL, low-density lipoprotein.

a Risk factors defined as current smoker, LDL ≥3.5 mmol/L, body mass index ≥25 kg/m^2^ or diabetes mellitus.

**Figure 1:**
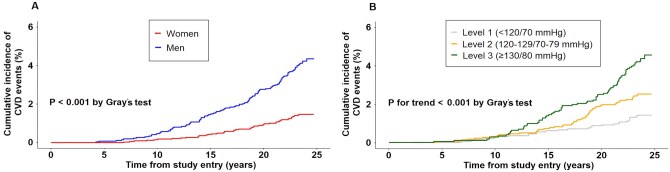
Cumulative incidence of cardiovascular disease events by sex (A) and by blood pressure levels (B). CVD, cardiovascular disease.

During a median follow-up time of 24 (24-24) years, a CVD event occurred in 158 individuals (2.7%), of which 48 (1.5%) were in women and 110 (4.2%) in men (Figure 1A). Table S1 shows the type of CVD events during follow-up.

### Association between BP and risk of CVD events (continuous data)

**Figure 2:**
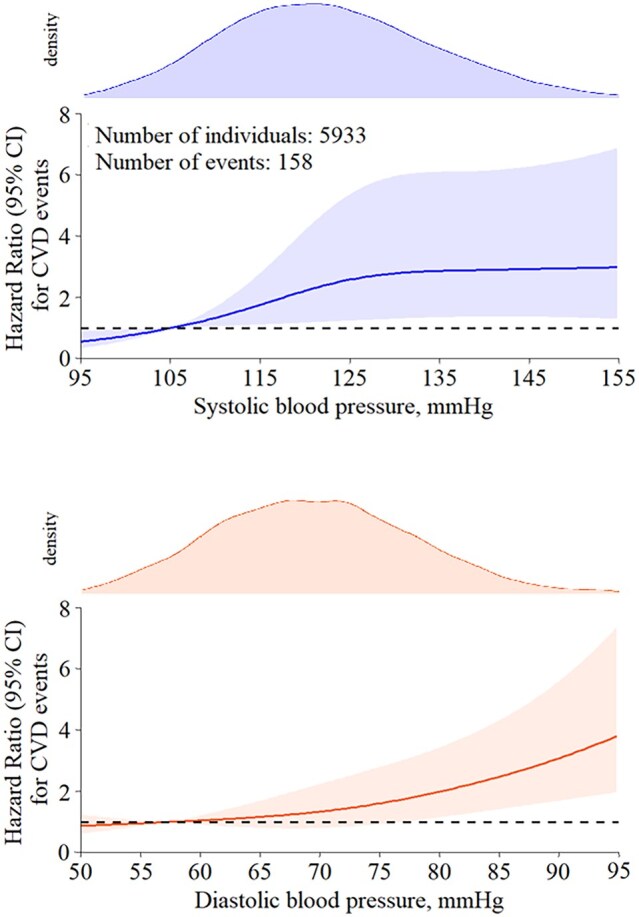
Risk of cardiovascular disease events as a function of systolic and diastolic blood pressure. Cox regression using restricted cubic splines. Reference was set at the 5th percentile, which was 105 and 56 mm Hg for systolic and diastolic BP, respectively. Solid lines indicate adjusted hazard ratios, and shadow shapes indicate 95% confidence intervals. For systolic and diastolic blood pressure, 34 and 31 participants were truncated, respectively. HR, hazard ratio; CI, confidence interval; CVD, cardiovascular disease.

The associations between systolic and diastolic BP and the risk of CVD events during long-term follow-up are shown in Figure 2 and Table S2. After multivariate adjustments, the HR for CVD events per 10 mm Hg higher systolic BP was 1.22 (95% confidence interval [CI]: 1.04-1.42). Per 5 mm Hg higher diastolic BP, the HR for CVD events was 1.20 (95% CI: 1.09-1.31). As opposed to diastolic BP, the HR of systolic BP in relation to CVD events reached a plateau at around 130 mm Hg.

### Association between BP and risk of CVD events (categorical data)

When the participants were divided into categories of BP, we observed a stepwise increase in CVD events between BP groups (Figure 1B). After multivariate adjustments, the HR between the BP categories level 3 vs level 1 was 1.90 (95% CI: 1.18-3.05).

### Sex-specific measurements of systolic and diastolic BP and risk of CVD events

**Figure 3:**
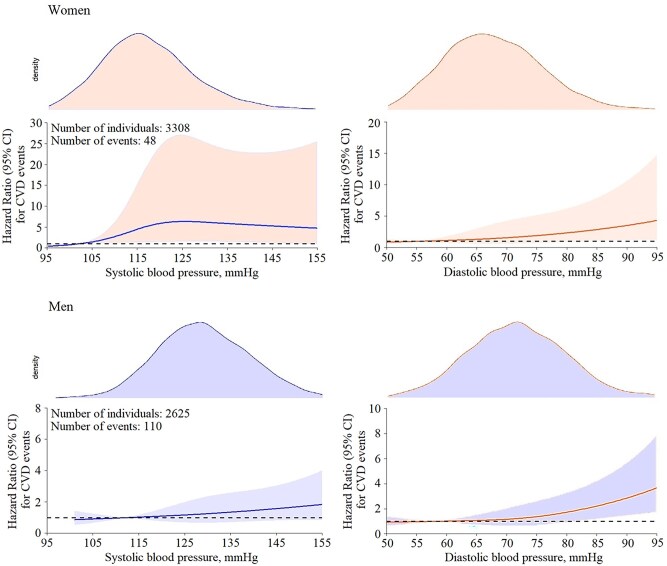
Cardiovascular disease events as functions of systolic and diastolic blood pressure in women and men separately. The distribution of blood pressure is presented as density plots. Cox regression using restricted cubic splines. 3 knots were placed at the 10th, 50th and 90th percentile. Reference was set at the 5th percentile. Solid lines indicate HRs, and shadow shapes indicate 95% CIs. For women, 4 and 11 participants were truncated for systolic and diastolic blood pressure, respectively; for men, the corresponding numbers were 30 and 20. HR, hazard ratio; CI, confidence interval; CVD, cardiovascular disease.

The median systolic and diastolic BP for women was 116 (110-123) and 67 (62-73) mm Hg, respectively. For men, systolic and diastolic BP were 128 (121-136) and 72 (66-77) mm Hg and higher compared to women (*P* < .001 for both). Amongst women and men, 48% and 15% had BP < 120/70 mm Hg, respectively. Baseline characteristics stratified by sex are shown in Table S3, and the population distributions of systolic and diastolic BP in women and men are presented in Figure 3.

In sex-stratified analyses, a comparable association between systolic BP and risk of CVD events was observed in women (HR = 1.30, 95% CI: 1.02-1.68) and men (HR = 1.15, 95% CI: 0.97-1.39) per 10 mm Hg increment. No statistically significant sex-interaction between systolic BP and risk of CVD events was found (*P* = .38), suggesting similar CVD risk of systolic BP for both women and men.

Furthermore, the association between diastolic BP (per 5 mm Hg increment) and CVD events in women and men was similar (HR = 1.20, 95% CI: 1.04-1.41 in women; HR 1.20, 95% CI: 1.08-1.34 in men).

### Components of BP and risk of CVD events

**Figure 4:**
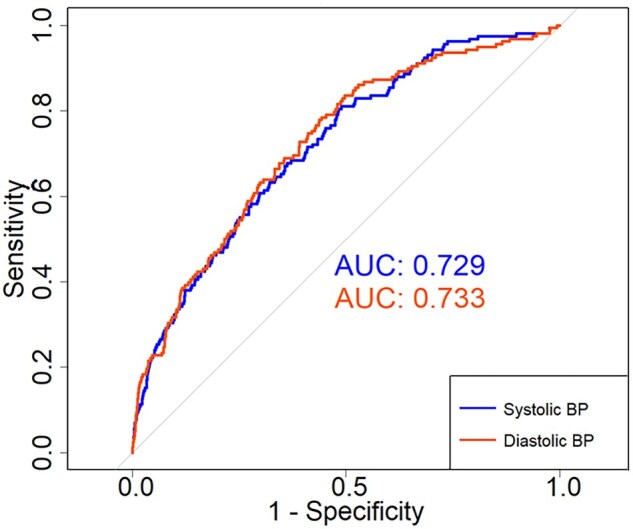
Receiver operating curves for systolic and diastolic blood pressure. AUC, area under the curve; BP, blood pressure.

When both systolic and diastolic BP were included in the same multivariate Cox model for the total population, the HR per 10 mm Hg higher systolic BP was 1.02 (95% CI: 0.84-1.24). The association between diastolic BP (per 5 mm Hg higher BP) and CVD events was, however, statistically significant (HR = 1.19, 95% CI: 1.06-1.33). The area under the curve (AUC) for a multivariate ROC including systolic BP was 0.729. When including diastolic instead of systolic BP, the AUC was 0.733 without an improvement in risk discrimination (*P* = .48) (Figure 4).

### Sensitivity analyses

In sensitivity analyses, the HR for the relation between BP and CVD events was unchanged for both systolic and diastolic BP when alcohol consumption, physical activity, use of antihypertensive medication, and heart rate were added (data not shown). Results were also unchanged when excluding missing values (Table S4). Figure S2 shows results stratified by the American College of Cardiology/American Heart Association classification, further dividing stage 1 hypertension into isolated systolic, isolated diastolic, and systolic-diastolic hypertension. In multivariate adjusted analyses, isolated diastolic stage 1 hypertension and stage 2 hypertension were associated with CVD events (Table S5).

## Discussion

In our contemporary cohort of 5933 young adults recruited from the general population at the age of 31, both systolic and diastolic BP were strongly associated with the risk of CVD events during 24 years of follow-up. Our study also displays differences in BP distributions between young women and men at the population level. Of note, 48% of women, but only 15% of men, had BP <120/70 mm Hg, consistent with “normal BP” by the European Society of Cardiology. In our study, the CVD risk associated with higher BP was similar in both sexes.

Our study provides novel contemporary data on the risk of BP as a CVD risk factor in young adults and expands previous knowledge. The historical cohorts, Framingham Heart Study and The Coronary Artery Risk Development in Young Adults (CARDIA) study,^23^^,^^24^ in addition to a subsequent meta-analysis, have previously demonstrated increased CVD risk with higher systolic and diastolic BP in young adults.^2^ However, many previous studies on BP and CVD events in young adults have typically included men only,^2^^,^^4^ have not adjusted for confounding CVD risk factors like total cholesterol or body mass index,^4^ or have not reported long-term follow-up after the age of 50 years,^25^ when the incidence of CVD events and prevalence of hypertension typically accelerate. Despite a substantial and steady BP decline in the Norwegian population,^15^ our findings demonstrate that BP remains a strong, independent CVD risk factor in young adults.

Some studies have reported a stronger association between BP and CVD risk in women compared with men, and advocated lower BP thresholds in women.^5^^,^^7^^,^^9^^,^^26^ However, one study from the UK Biobank showing BP being a stronger CVD risk factor in women did not adjust its models for total cholesterol,^7^ and a recent, more refined study from the same cohort did not support these findings.^27^ Others have typically not performed sex-interaction testing when reporting that higher BP is a stronger CVD risk factor in women.^26^ Another study of middle-aged individuals included a selected cohort of patients admitted with acute coronary syndrome, of whom a high proportion had unstable angina.^5^ In our study, we also observed an apparently stronger association between systolic BP and CVD risk in women, although the hazard ratio estimates were comparable. However, interaction testing was not statistically significant, and the sex-specific distribution of BP values, in which women generally had lower BP (and few men had BP <120/70 mm Hg), likely accounted for this finding, rather than systolic BP being a truly stronger risk factor for CVD in young women than in men. Our findings are consistent with the recent UK Biobank study, which used absolute risk differences and relative risk estimates as well as a reference group, and showed that the blood pressure level at which hypertension is defined should not vary by sex.^27^ Our results also demonstrate the favorable CVD prognosis in participants with BP <120/70 mm Hg, which was present in nearly half of the women at baseline, and was subsequently associated with a low rate of CVD events during long-term follow-up.

The BP component that best captures CVD risk may vary by age and type of event. In our study, systolic and diastolic BP showed similar risk discrimination for CVD events. However, only isolated diastolic stage 1 hypertension and stage 2 hypertension were associated with CVD events with normal BP as reference. This finding suggests that interpretation of systolic and diastolic BP separately may be important for individual risk stratification, with particular emphasis on high diastolic BP. In young adults, elevated diastolic blood pressure may be related to increased vascular resistance due to sympathetic nervous system activation, which, over time, can lead to alterations in vascular function.^28^^,^^29^ With increasing arterial stiffness and reduced compliance occurring later in life, isolated systolic hypertension or pulse pressure may serve as better predictors of CVD events.^30^

There is a growing recognition within the cardiovascular community of the need to extend the preventive horizon beyond the traditional 10-year framework.^31^^,^^32^ Young adulthood may represent a critical window for improved primary prevention, before the fibrotic and atherosclerotic processes have progressed to irreversible stages and clinical events are likely to occur.^33^ Our findings confirm BP as an independent CVD risk factor in a contemporary cohort and support the inclusion of BP in early risk assessment for both women and men, alongside other traditional risk factors.

Our results also demonstrate an opportunity to select individuals at the highest CVD risk who may potentially benefit from pharmacological antihypertensive treatment, already at the age of 30 years. We suggest that repeated office measurements, use of ambulatory and/or home BP monitoring, searching for hypertension mediated organ damage (especially microalbuminuria and left ventricular hypertrophy), assessment for secondary causes of hypertension and a broad investigation of other CVD risk factors (smoking, family history, dyslipidemia, diabetes mellitus, overweight, unhealthy diet including high salt intake, physical inactivity) should be addressed when encountering BP values in the hypertensive range in young adults. In the future, polygenic risk scores, biomarkers, quantification of subclinical fibrosis and/or atherosclerosis, or the use of artificial intelligence may help clinicians to select young adults who are even more likely to benefit from pharmacological antihypertensive treatment, but there is no clear evidence supporting these strategies as of today.

### Limitations

Residual confounding cannot be excluded, although we have adjusted for the most important CVD risk factors at baseline. We did not assess hypertensive phenotypes or white-coat hypertension, which are frequent among young adults. Thus, we may have overestimated BP values, and our results may therefore be biased toward lower risk estimates. We lack information regarding antihypertensive prescriptions during follow-up, which may have led to further underestimation of the risk associated with BP, especially in participants with BP > 140/90 mm Hg. The CVD events were recorded from national registries, which may represent a potential source of error. Event rates were low in our young cohort, as expected, and our study may have been underpowered to show potential sex differences of BP as a CVD risk factor, particularly among women with BP > 140/90 mm Hg.

Finally, though prospective, our study had an observational design, which does not allow conclusions regarding causality. While BP has been established as a causal CVD risk factor in numerous clinical trials (mostly in high-risk patients), and a register-based study has demonstrated that adherence to antihypertensive treatment is associated with improved outcomes in young adults,^34^ we believe that BP-lowering intervention trials targeting BP reduction in young adults are unlikely to be conducted.

## Conclusions

In our prospective contemporary cohort of 5933 young adults recruited from the general population, with 24 years of follow-up, systolic and diastolic BP were strongly related to future CVD events with no sex differences. Our data suggest that BP should be accounted for in the risk assessment of young adults, equally among women and men.

## Supplementary Material

hpag025_Supplementary_Data

## Data Availability

The Norwegian Data Protection Agency does not allow open access to our data. Upon reasonable request, additional data can be made available through a request to the corresponding author, pending approval from Norwegian authorities.
