# Supplementary material for: Blood pressure and cardiovascular risk in a contemporary cohort of young adults
Source: Am J Hypertens. 2026 Mar 25;39(8):1005–12. doi: 10.1093/ajh/hpag025 (PMC13369950; doi:10.1093/ajh/hpag025)
Supplement: hpag025_Supplementary_Data [file hpag025_supplementary_data.docx]

**Supplementary Material**

**Table S1. Type of first cardiovascular disease events during follow-up**

| **Type of event** | **Overall**  **n=5933** | **Women**  **n=3308** | **Men**  **n=2625** | **International classification of Disease 10 codes** |
| --- | --- | --- | --- | --- |
| Cardiovascular death | 11 | 2 | 9 | Death due to any of the codes listed below. In addition, I46 and R99 |
| Non-fatal myocardial infarction | 67 | 18 | 49 | I21-I22 |
| Non-fatal cerebral stroke | 36 | 15 | 21 | I63-I64, I67 |
| Non-fatal cerebral hemorrhage | 11 | 5 | 6 | I60-I61 |
| Hospitalization for heart failure | 10 | 4 | 6 | I50 |
| Coronary revascularization | 23 | 4 | 19 | Defined by the procedural codes FNA, FNC, FNE12B, FNP02B and FNQ05B when linked to I20 or I25 |
| Total | 158 | 48 | 110 |  |

Values are numbers

**Table S2. Univariate and multivariate adjusted HRs**

|  | Univariate adjusted | |  | Multivariate adjusted | |
| --- | --- | --- | --- | --- | --- |
|  | HR (95% CI) | P-value |  | HR (95% CI) | P-value |
| Systolic BP, per 10 mmHg increment | 1.47 (1.30–1.65) | P < 0.001 |  | 1.22 (1.05-1.42) | P = 0.0089 |
| Diastolic BP, per 5 mmHg increment | 1.32 (1.22–1.43) | P < 0.001 |  | 1.20 (1.09-1.31) | P < 0.001 |

Multivariate adjustments for sex, non-western ancestry, education of less than 12 years, smoking status, diabetes mellitus, body mass index and total cholesterol. BP, blood pressure; CI, confidence interval; HR, hazard ratio.

**Table S3. Baseline characteristics stratified by sex (Overall included in Table 1)**

| **Variables** | **Women** | **Men** |
| --- | --- | --- |
| Number of individuals, n (%) | 3308 (56) | 2625 (44) |
| Age, years | 31 (30-31) | 31 (30-31) |
| Non-western ancestry, n (%) | 471 (14) | 341 (13) |
| Current smokers, n (%) | 780 (24) | 601 (23) |
| Diabetes, n (%) | 29 (1) | 15 (1) |
| Education, years | 16 (13-18) | 16 (13-18) |
| Pregnant (self-reported), n (%) | 191 (6) |  |
| Body mass index, kg/m^2^ | 23 (21-26) | 25 (23-27) |
| Systolic BP, mmHg | 116 (110-123) | 128 (121-136) |
| Diastolic BP, mmHg | 67 (62-73) | 72 (66-77) |
| Use of antihypertensives, n (%) | 39 (1) | 31 (1) |
| Level 1 BP category ^a^, n (%) | 1595 (48) | 385 (15) |
| Level 2 BP category ^b^, n (%) | 1199 (36) | 1013 (39) |
| Level 3 BP category ^c^, n (%) | 514 (16) | 1227 (47) |
| Total cholesterol, mmol/L | 4.9 (4.3-5.5) | 5.0 (4.5-5.7) |
| LDL cholesterol, mmol/L | 2.8 (2.4-3.3) | 3.1 (2.6-3.7) |
| HDL cholesterol, mmol/L | 1.5 (1.3-1.8) | 1.2 (1.0-1.4) |
| Plasma triglycerides, mmol/L | 0.9 (0.7-1.3) | 1.5 (1.0-2.2) |
| Without traditional risk factors^d^ | 1421 (44) | 748 (29) |
| Without traditional risk factors^d^ and Level 1 BP | 766 (49) | 161 (42) |
| Without traditional risk factors^d^ and Level 2 BP | 476 (40) | 323 (32) |
| Without traditional risk factors^d^ and Level 3 BP | 179 (36) | 264 (22) |

Values are median (interquartile range) for continuous variables and number (%) for categorical variables

HDL, high density lipoprotein; LDL, low density lipoprotein; BP, blood pressure

^a^ Defined as systolic blood pressure <120 mmHg and diastolic blood pressure <70 mmHg

^b^ Defined as systolic blood pressure 120-129 mmHg or diastolic blood pressure 70-79 mmHg

^c^ Defined as systolic blood pressure ≥130 mmHg or diastolic blood pressure ≥80 mmHg

^d^ Risk factors defined as current smoker, LDL ≥3.5 mmol/L, body mass index ≥25 kg/m^2^ or diabetes mellitus

**Table S4.** HRs for systolic and diastolic BP when excluding missing values

|  | Number of individuals | Number of events | HR (95% CI) |
| --- | --- | --- | --- |
| Systolic BP, per 10 mmHg increment | 5692 | 148 | 1.23 (1.05-1.44) |
| Diastolic BP, per 5 mmHg increment | 5692 | 148 | 1.18 (1.07-1.30) |

**Table S5. Univariate and multivariate adjusted HR using the ACC/AHA classification of BP also including isolated systolic and isolated diastolic hypertension**

| Univariate analyses | Number of individuals | Number of events | HR (95% CI) |
| --- | --- | --- | --- |
| Normal BP | 2528 | 35 | Reference |
| Elevated BP | 1664 | 48 | 2.11 (1.37-2.62) |
| Stage 1 isolated systolic hypertension | 792 | 27 | 2.50 (1.51-4.13) |
| Stage 1 isolated diastolic hypertension | 191 | 10 | 3.86 (1.91-7.79) |
| Stage 1 systolic and diastolic hypertension | 234 | 9 | 2.83 (1.36-5.89) |
| Stage 2 hypertension | 524 | 29 | 4.09 (2.50-6.69) |
|  |  |  |  |
| Multivariate adjusted analyses |  |  |  |
| Normal BP | 2528 | 35 | Reference |
| Elevated BP | 1664 | 48 | 1.53 (0.96-2.43) |
| Stage 1 isolated systolic hypertension | 792 | 27 | 1.52 (0.87-2.64) |
| Stage 1 isolated diastolic hypertension | 191 | 10 | 2.89 (1.41-5.93) |
| Stage 1 systolic and diastolic hypertension | 234 | 9 | 1.50 (0.70-3.23) |
| Stage 2 hypertension | 524 | 29 | 2.09 (1.84-2.69) |

Normal BP is defined as < 120/80 mmHg, elevated BP 120-129/<80 mmHg, stage 1 isolated systolic hypertension < 130-139/80-89 mmHg, stage 1 isolated diastolic hypertension <130/80-89 mmHg, stage 1 systolic and diastolic hypertension 130-139/80-89 mmHg and stage 2 hypertension $\geq$ 140/90 mmHg. Multivariate cox-regression models adjusted for sex, non-western ancestry, education of less than 12 years, smoking status, diabetes mellitus, body mass index and total cholesterol. ACC/AHA, American College of Cardiology/American Heart Association; BP, blood pressure; HR, hazard ratio

**Figure S1. Cohort creation chart**


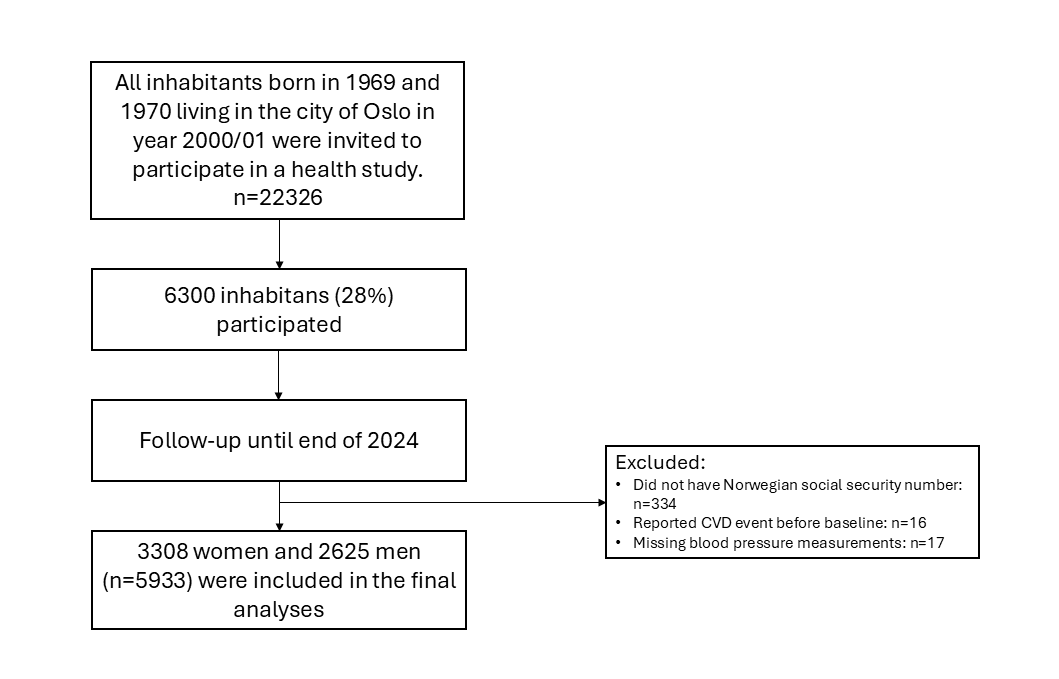


**Figure S2. Cumulative incidence of cardiovascular disease events grouped by the American College of Cardiology/American Heart Association classification with stratification of stage 1 hypertension into isolated systolic, isolated diastolic and systolic-diastolic hypertension.**


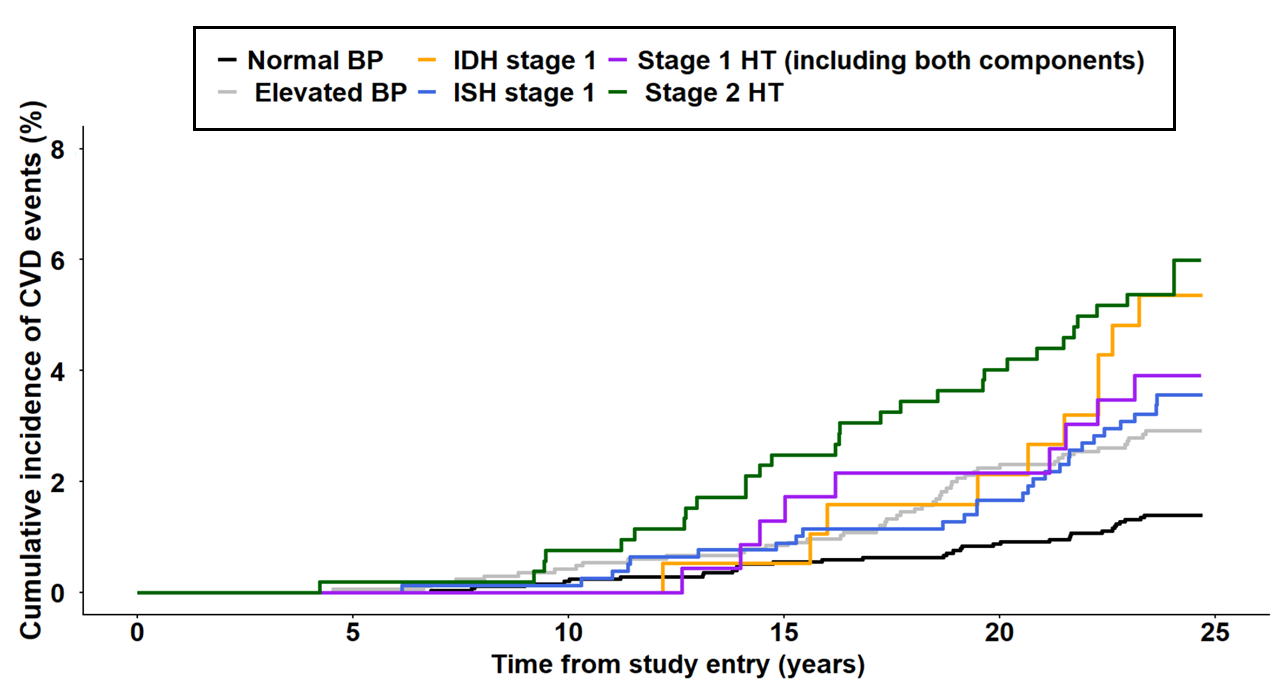


Normal BP is defined as < 120/80 mmHg, elevated BP 120-129/<80 mmHg, isolated systolic hypertension stage 1 < 130-139/80-89 mmHg, isolated diastolic hypertension stage 1 <130/80-89 mmHg, stage 1 systolic and diastolic hypertension 130-139/80-89 mmHg and stage 2 hypertension $\geq$ 140/90 mmHg. BP, blood pressure; HT, hypertension; IDH, isolated diastolic hypertension; ISH, isolated systolic hypertension.
